# 细胞印迹技术在生物医学领域的应用进展

**DOI:** 10.3724/SP.J.1123.2025.04007

**Published:** 2026-01-08

**Authors:** Xinmiao ZHAO, Zhiyuan ZHANG, Wenjing SUN, Guangyan QING

**Affiliations:** 1. 辽宁师范大学化学化工学院，辽宁 大连 116029; 1. School of Chemistry and Chemical Engineering，Liaoning Normal University，Dalian 116029，China; 2. 中国科学院大连化学物理研究所，辽宁 大连 116023; 2. Dalian Institute of Chemical Physics，Chinese Academy of Sciences，Dalian 116023，China; 3. 江南大学生命科学与健康工程学院，江苏 无锡 214122; 3. School of Life Science and Health Engineering，Jiangnan University，Wuxi 214122，China

**Keywords:** 细胞印迹, 细胞捕获, 细胞识别与分选, 细胞培养, 生物传感器, cell imprinting, cell capture, cell recognition and sorting, cell culture, biosensor

## Abstract

细胞印迹技术作为实现细胞特异性识别领域的关键技术，依靠其对细胞表面抗原、受体等生物标志物的精准识别能力，已成为现代生物医学研究的重要基石。该技术不仅广泛应用于疾病标志物检测、细胞功能与行为机制研究等基础领域，更在稀有细胞分离、靶向药物开发等前沿应用中发挥着关键作用，持续推动着生命科学研究的创新发展。虽然该技术已取得显著进展，但仍面临着若干亟待解决的问题，具体包括：印迹工艺的优化（提升抗干扰性和保真性）及适配复杂生物环境新方法的开发。针对这些问题，研究人员通过创新材料设计与工艺优化，近期在多个领域取得了突破性进展：首先，在稀有细胞捕获方面，该技术显著提升了复杂样本中关键稀有细胞的分离效率，为癌症早期诊断和血液病研究提供了高效的解决方案；其次，在细胞培养领域，其独特的印迹表面可精确调控细胞黏附蛋白表达，优化体外培养微环境；最后，在生物传感领域，基于该技术开发的传感器展现出卓越的灵敏度和特异性，为疾病监测开辟了新途径。本文系统梳理了细胞印迹技术的研究现状与发展动态，深入剖析了其技术瓶颈与突破方向，旨在为相关研究提供全面的理论参考，进一步促进该技术在生物医学领域的创新应用与跨越式发展。

## Abstract (en)

Cell imprinting technology， as a key technique in the field of cell-specific recognition， has become an important cornerstone of modern biomedical research. It relies on its precise recognition capability for biomarkers such as cell surface antigens and receptors. This technology is not only widely applied in basic fields， including disease marker detection and research on cell function and behavioral mechanisms， but also plays a crucial role in frontier applications like rare cell isolation and targeted drug development. It continuously promotes the innovative development of life science research. Although significant progress has been made in this technology， it still faces several urgent challenges. These specifically include optimization of the imprinting process （to enhance anti-interference ability and fidelity） and development of new methods adapted to complex biological environments. In response to these issues， researchers have achieved breakthrough progress in multiple fields through innovative material design and process optimization recently. First， in rare cell capture， this technology has significantly improved the separation efficiency of key rare cells in complex samples， providing an efficient solution for early cancer diagnosis and blood disease research. Second， in the field of cell culture， its unique imprinted surface can precisely regulate the expression of cell adhesion proteins to optimize the in vitro culture microenvironment. Finally， in biosensing， sensors developed based on this technology have demonstrated excellent sensitivity and specificity， opening up new avenues for disease monitoring. This paper systematically reviews the research status and development trends of cell imprinting technology， deeply analyzes its technical bottlenecks and breakthrough directions. The aim is to provide a comprehensive theoretical reference for related research and further promote the innovative application and leapfrog development of this technology in the biomedical field.

在色谱分析领域，分子印迹技术因其独特的技术优势已成为前沿研究热点。该技术通过在聚合物基质中构建能精准匹配模板分子尺寸、构象及官能团空间排布的人工识别位点，实现对目标分子的精准识别^［1］^。凭借这种高度特异性的识别能力，该技术在复杂样品分离与检测等应用中脱颖而出，有效解决了传统色谱中选择性不足的难题^［2］^。然而，当其应用于复杂生物体系中完整细胞的分析时，分子印迹技术暴露出显著的短板——其针对小分子设计的识别位点难以适配细胞的三维复杂结构，无法有效识别细胞表面多种特异性抗原或标志物，并且在活体样本分析中易受生物基质的干扰。这些技术瓶颈促使人们将目光投向细胞印迹技术，该技术运用创新的印迹策略，以完整的细胞为模板构建识别体系^［3］^，为色谱分析领域开辟了新的研究方向和技术突破路径。

细胞印迹技术的模板制备策略分为细胞膜-分子印迹和全细胞印迹两类^［4］^。细胞膜-分子印迹技术通过利用目标细胞表面特异性分子与聚合物的结合^［5-8］^，实现对目标细胞的高特异性识别。例如，Zhou等^［9］^基于磷脂和分子印迹技术，成功制备了磷脂酰丝氨酸（phosphatidylserine， PS）和鞘磷脂（sphingomyelin， SM）分子印迹聚合物，在细胞膜选择性识别、凋亡细胞成像及外泌体分离等领域取得了突破性进展。此外，基于膜蛋白^［10］^和糖链^［11］^的细胞膜-分子印迹技术也迅速发展，这些技术通过对膜蛋白和糖链特征结构的精准捕捉，为疾病诊断与药物研发开拓了新路径。然而，这项技术存在一定的局限性：当细胞表面标志物呈现显著的动态变化特征，或存在细胞群间的异质性差异时，分子印迹聚合物的特异性和亲和力会显著下降，进而引发识别偏差，降低检测效率。为解决这一问题，全细胞印迹技术应运而生。该技术以完整细胞（如红细胞、癌细胞）为模板，选用聚苯乙烯（polystyrene， PS）、聚二甲基硅氧烷（polydimethylsiloxane， PDMS）等柔性聚合物为基底，借助软光刻、微接触印刷等物理手段^［12］^，在基底表面构建与细胞形态高度匹配的仿生微纳结构^［13］^。值得注意的是，单纯依靠形貌匹配产生的物理作用难以产生细胞与印迹模板之间的强亲和力。因此，研究人员常通过特异性抗原的结合作用对细胞进行固定^［14，15］^。全细胞印迹技术通过细胞与印迹模板间物理形貌的匹配和化学位点的识别^［16］^，显著地提升了对目标细胞的识别效率与结合的稳定性，为细胞印迹技术的发展开辟了全新路径。

全细胞印迹技术的核心优势在于其独特的仿生结构与分子识别能力，该优势有效克服了传统技术依赖抗体的局限性。该技术在多个领域展现出重要的应用价值：在生物医学领域，可利用该技术高效地捕获血液中的肝癌细胞，进而辅助癌症的早期筛查与病情的监测工作，推动精准医学的发展^［17］^；在细胞生物学研究中，该技术为深入解析细胞间相互作用机制及细胞-材料界面行为提供了有力工具^［18］^；在生物传感领域，基于该技术构建高灵敏度和高特异性的细胞传感器，实现了对特定细胞的快速和准确检测^［19］^。这些优势使其在疾病防控、环境监测等诸多方面展现出极为可观的应用潜力，成为推动相关领域不断向前发展的重要力量。本综述重点讲述了全细胞印迹技术在生物医学领域的应用进展，着重探讨了全细胞印迹技术现存的挑战及未来的发展趋势。

## 1 全细胞印迹技术使用的策略

全细胞印迹制备策略根据应用需求不同^［20］^可以分成直接印迹和间接印迹两大类方法^［21］^。直接印迹法主要包括印章印迹、薄膜印迹和牺牲层印迹3种技术。其中印章印迹是一种操作简洁、应用广泛的印迹方法，主要步骤是将目标细胞铺在聚合物表面进行压印，之后洗去模板细胞，形成印迹空腔；薄膜印迹适用于印迹脆弱的生物样品（如细胞），模板细胞可以直接放置在有支撑载体的聚合物表面，待模板细胞在聚合物表面自然沉降，留下印迹痕迹；牺牲层印迹法是在样品和聚合物之间添加与聚合物共价结合的牺牲层，在防止样品与聚合物发生反应的基础上引入新的官能团，便于去除模板，实现多位点结合。间接印迹法主要包括人工模板印迹和人工抗体复制品印迹两种方法，其中人工模板印迹常常用于生长过程中形态不稳定的细胞或是实验人员不便接触的致病菌，它的制备方法是将目标细胞先压印在柔软的聚合物表面，去除细胞模板后，再用另外一种聚合物压印在第一块聚合物表面，从而获得人工模板^［22］^，该模板能够重复使用，且成熟的人工模板印迹可以代替真实的目标细胞；人工抗体复制品印迹^［23，24］^利用天然抗体和目标细胞间能够特异性结合的性质，将天然抗体与聚合物共混，剥离天然抗体留下可与目标细胞特异性结合的印迹空腔，对目标细胞具有极高的选择性与特异性，也可以重复使用，但该方法需要分析目标细胞的特异性抗体，相对于亚结构印迹，这一方法在前期准备更加复杂。全细胞印迹技术凭借其独特的发展历程和多样化的印迹方法，在技术原理与实现路径上不断革新突破，为应对生物医学与材料科学领域的各种挑战奠定了坚实基础。

## 2 全细胞印迹技术的应用

随着全细胞印迹技术的日趋成熟，其研究重心已从基础方法开发转向实际应用探索，在稀有细胞捕获、单细胞分选、细胞培养、生物传感等关键领域展现出巨大潜力，由此开启了全细胞印迹技术广泛应用的新篇章。

### 2.1 稀有细胞捕获

稀有细胞是指在复杂生物样本中含量极低的特殊细胞群体，主要包括循环肿瘤细胞（circulating tumor cell， CTC）、多功能干细胞等^［25］^。这类细胞在疾病诊断和再生医学等领域具有重要的临床价值。以CTC为例，这类细胞从原发肿瘤上脱落，进入血液循环系统。通过对CTC的检测，不仅能实时跟踪肿瘤的发展与转移情况，助力癌症的早期诊断，还能为预后评估提供依据，推动个性化治疗方案的制定^［26，27］^。当前稀有细胞捕获方法可分为物理法和生物亲和法：前者基于细胞的物理特性（如大小、密度、电荷）分离^［28，29］^，后者依赖抗体与表面抗原结合及荧光标记鉴定。全细胞印迹技术作为新兴的印迹技术，以目标细胞为模板，在聚合物基质中构建与目标细胞表面形貌和化学性质高度互补的三维识别位点，实现高特异性捕获。相较于传统方法，其优势在于成本低、稳定性高、材料可重复使用，且在复杂样本中仍保持精准性，为癌症检测等提供高效的解决方案。

Liu等^［30-32］^通过三阶段的技术迭代，开发出了可以高效捕获稀有细胞的平台，助力癌症早期诊断与治疗监测（图1）。研究人员将全细胞印迹技术与点击化学结合，开发适配体功能化全细胞印迹基底（cell-imprinted substrate with aptamer functionalization， APT-CIS）材料^［30］^，以多聚甲醛固定人肝癌细胞（SMMC-7721），通过PDMS固化形成形貌匹配的微纳印迹腔，通过硫醇-烯点击化学（thiol-ene click chemistry）将丙烯酸酯和荧光标记的适体共价连接到含有硫醇基的细胞印迹底物（SH-cell-imprinted substrate， SH-CIS）表面，特异性识别SMMC-7721细胞，效率达93.9%±0.8%（图1a）。为进一步提升复杂生物环境下的捕获效率，Liu等^［31］^设计出位点定向修饰的适配体水凝胶（cell-imprinted hydrogel with site-directed modification of aptamers， APT-CIH）。将丙烯酰胺、*N*， *N′*-亚甲基双丙烯酰胺与三功能可切割交联剂（trifunctional cleavable cross-linker， TCC）的甲基丙烯酰基团共聚，形成覆盖细胞表面的水凝胶层，并在印迹位点引入多价适体，增强化学亲和性。APT-CIH对SMMC-7721细胞的捕获效率达94.7%±0.9%，和之前的工作相比，通过TCC实现适体位点定向修饰（而非随机分布），形成局部高密度适体簇，增强了结合强度（图1b）。为攻克捕获细胞活性保持与无损释放的瓶颈，Liu等^［32］^紧接着开发了具有全细胞印迹与硼酸亲和协同效应的水凝胶（cell-imprinted hydrogel with 3-acrylamido phenylboronic acid， PBA-CIH）。用丙烯酰化-3-氨基苯硼酸（3-acrylamido phenylboronic acid， 3-AAPBA）修饰的印迹网络，利用硼酸与唾液酸的可逆结合实现细胞表面糖蛋白的捕获。相比适体或点击化学，这种方法更适用于唾液酸高表达的肿瘤细胞（图1c）。可在13例肝癌患者中成功捕获CTC（5~20个/mL），通过单细胞测序揭示肿瘤异质性，指导个性化治疗。从APT-CIS到PBA-CIH的技术演进，实现了化学协同识别与动态释放的创新跨越。

**图1:**
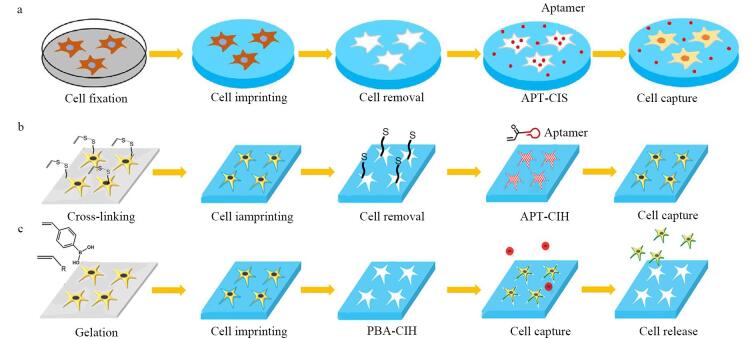
SMMC-7721细胞的捕获与释放流程图^［30-32］^

Lv等^［33］^基于金纳米棒近红外光热效应与热敏水凝胶的协同作用，设计了一种近红外光响应性水凝胶（图2），为CTC的捕获与位点释放带来了新突破。在实验过程中，该水凝胶对人乳腺癌（michigan cancer foundation-7， MCF-7）细胞展现出优异的捕获性能。在体外实验中，当用抗上皮细胞黏附分子（anti-epithelial cell adhesion molecule， anti-EpCAM）修饰的水凝胶捕获细胞时，对MCF-7细胞的捕获效率可达92%±6%（未明确细胞数量和孵育面积相关条件）。通过温度响应释放和光热选择性释放两种方式，分别有95%±4%和92%±6%的捕获细胞能被释放，释放细胞的活力分别为95%和90%，且释放的细胞可在体外直接培养和增殖，表明其增殖能力未受影响。在存在不同类型干扰细胞（如HeLa细胞和白细胞）的情况下，该水凝胶对MCF-7细胞的捕获效率远高于干扰细胞，显示出良好的特异性。最重要的是，将MCF-7细胞掺入全血样本模拟临床情况时，该水凝胶的捕获效率为52%，与其他静态CTC检测装置相当，且能通过三色免疫细胞化学方法准确识别捕获的细胞，这表明其在复杂临床样本中捕获CTC的可行性。在实际临床应用潜力评估中，将该水凝胶应用于13位患者全血样本中CTC的检测，成功捕获并检测到患者血液中的CTC，且在捕获效率上优于anti-EpCAM修饰的光滑明胶底物，还能通过近红外光实现单个CTC的位点特异性释放并进行基因分析，为研究肿瘤异质性的分子机制提供了可能，为个性化抗肿瘤治疗开辟了新途径。

**图2:**
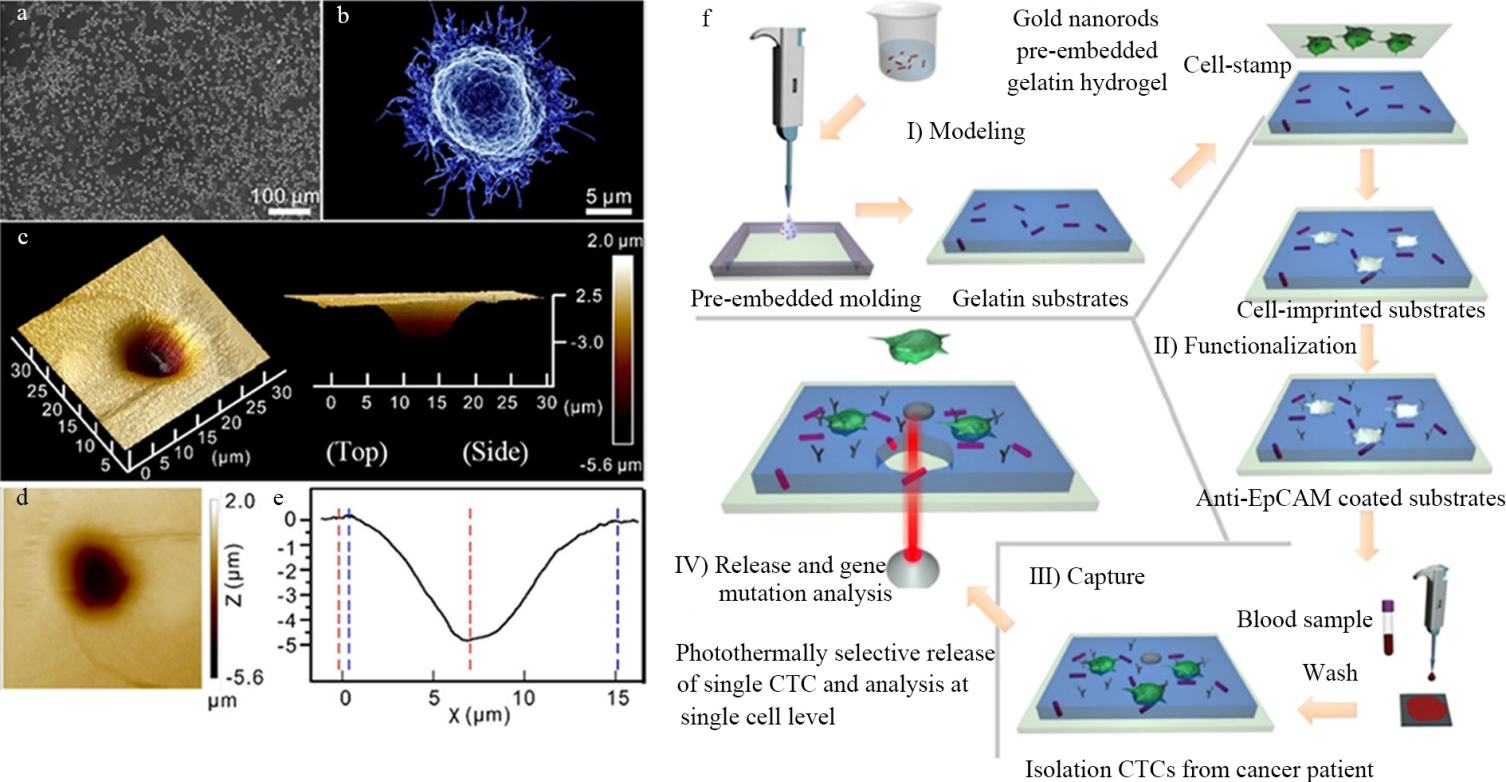
近红外光响应性水凝胶捕获与释放CTC流程图^［33］^

### 2.2 单细胞分选

在生物医学研究中单细胞分选是细胞疗法设计和制造的强大手段^［34］^。该技术精确分离特定单细胞，有助于全面了解细胞信息，如肿瘤细胞亚群分析、血型检测、外泌体收集等^［35-37］^，还可以通过获取纯净细胞样本用于基因测序、蛋白质组学研究，为疾病诊断、药物研发提供关键信息，推动精准医学发展。

Sun等^［38］^开发了一种新型酶联免疫吸附测定（enzyme linked immunosorbent assay， ELISA）的三明治策略，通过整合全细胞印迹基底（cell-imprinted substrate， CIS）与pH敏感变色的石墨烯氧化物（allochroic-graphene oxide， AGO），实现了肝细胞癌（hepatocellular carcinoma， HCC）细胞的高特异性可视化分选（图3）。CIS采用PDMS构建，通过固定模板细胞（如HLE肝癌细胞）并固化形成形貌匹配的微米级印迹腔（直径约22 µm），结合表面化学识别位点，对目标细胞捕获效率达97.5%，且对非靶细胞的交叉捕获率低至12%。AGO通过将苯硼酸修饰至氧化石墨烯表面，并负载pH敏感指示剂酚酞（phenolphthalein， PP），可在生理pH下特异性结合细胞表面糖蛋白的顺式二醇基团，在碱性条件下触发PP释放显色，实现信号的放大与可视化输出。在复杂肝细胞悬液（含红细胞）中，CIS连接的一氧化石墨烯对低浓度HLE肝癌细胞（1×10⁵ 个/mL）的回收率达80.67%±4.33%，且对非靶细胞（如AML12正常肝细胞）的误捕率仅为2.4%±1.6%。该技术通过“形貌-化学”双层级识别机制，有效避免了传统抗体依赖方法中成本高、稳定性差等缺陷。实验表明，CIS的印迹腔密度与细胞捕获效率呈正相关，而AGO的硼酸亲和识别在生理条件下即可完成，显著提升了临床实用性。此外，该方法操作简便（总耗时<5 h）、可重复使用（材料效率衰减<8%），为CTC分选及异质性研究提供了高效工具，有望推动个体化治疗方案的精准制定。

**图3:**
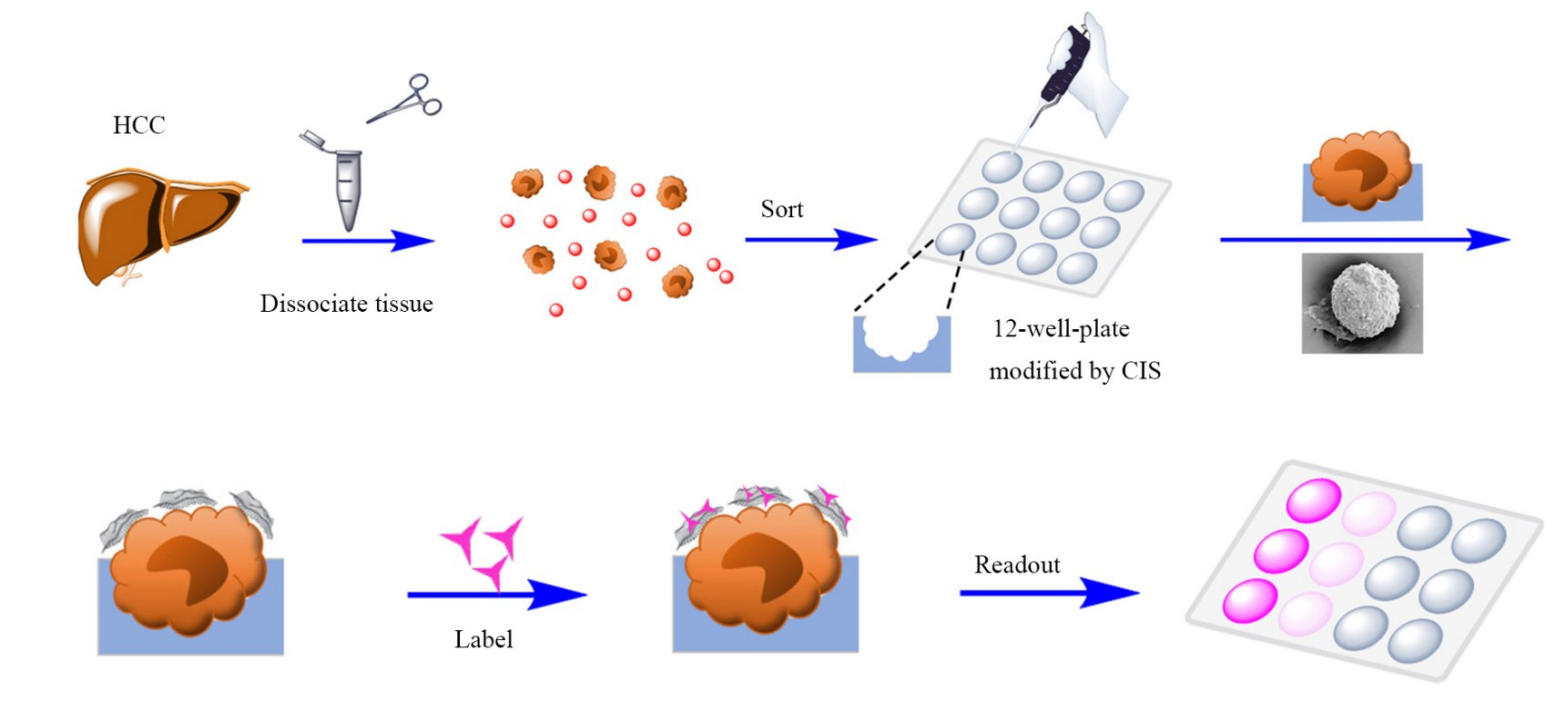
CIS连接的一氧化石墨烯用于细胞测定的机制^［38］^

### 2.3 细胞培养

近年来，全细胞印迹技术逐渐运用在细胞培养领域。细胞培养在生物医学研究中意义非凡，作为现代生命科学研究的基础工具，细胞培养为探索细胞生理病理过程提供了关键的体外模型系统。该技术有助于推动药物研发、疾病机制探究及细胞治疗等方面的发展，是现代生命科学不可或缺的工具。

Kashani等^［39］^针对体外细胞培养环境与体内生理条件存在显著差异这一关键问题，创造性地通过微流体芯片制作了CIS，提升了传统全细胞印迹方法的效率，改进了干细胞分化诱导的策略。在这项研究中，他们借助微流控芯片设计与制备技术，成功制作出软骨全细胞印迹基底（图4）。设计了与软骨细胞尺寸相当的微通道来固定软骨细胞的位置，并且不影响培养液的交换。研究人员将含有软骨细胞的溶液注入其中，使软骨细胞在芯片微通道内排列成特定图案，经固定、PDMS铸型等步骤，获得具有软骨细胞图案的印迹基底。随后，把脂肪来源的间充质干细胞（adipose-derived mesenchymal stem cells， ADSCs）接种到该全细胞印迹基底上进行培养。在培养过程中，当ADSCs通过精心设计的微流控系统时，能精确地定位在软骨细胞图案上。在不使用任何化学生长因子的情况下，经过14天的培养，ADSCs由成纤维细胞样的形态逐渐转变为软骨细胞的球形形态。通过免疫染色和基因表达分析等多种检测手段进一步证实，细胞的软骨分化得到了显著改善。例如，基因表达分析显示，与未分化的干细胞相比，在全细胞印迹基底上培养的ADSCs中软骨特异性基因标志物（如胶原蛋白Ⅱ）的表达显著增加。研究人员还进行了兔模型体内实验，将在全细胞印迹基底上分化的ADSCs接种到胶原蛋白-透明质酸支架上，然后移植到兔关节软骨缺损处，6个月后与对照组相比后发现接受分化干细胞移植的实验组关节软骨缺损得到了成功再生。这一成果充分展示了全细胞印迹技术在细胞培养领域的巨大潜力，为未来临床治疗软骨损伤等相关疾病提供了一种安全、有效的新策略，有望推动组织工程和再生医学的进一步发展。

**图4:**
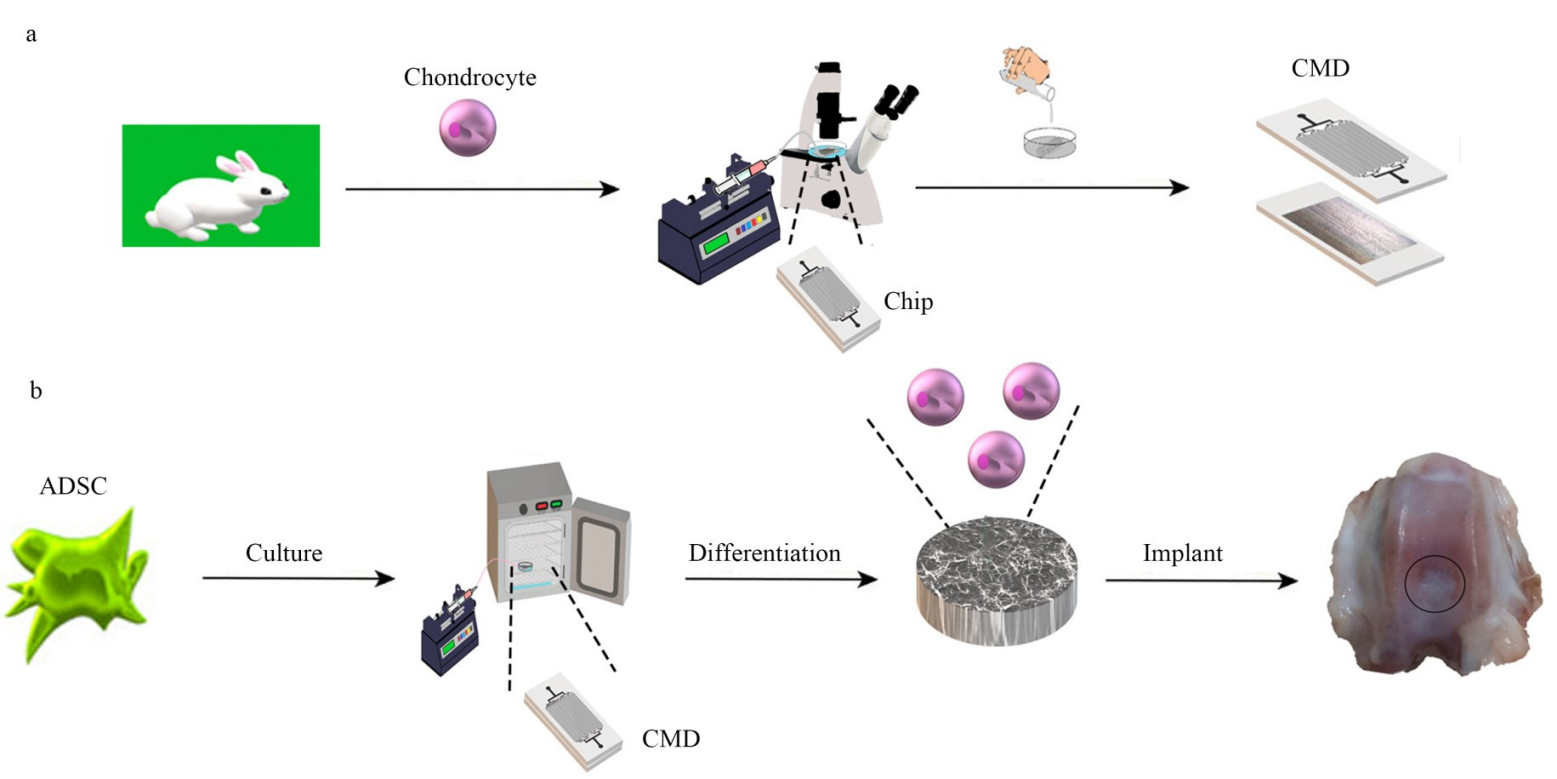
基于CIS的干细胞分化集成微流控装置图^［39］^

此外，Mashinchian等^［40］^以成熟的人类角质形成细胞为模板制备全细胞印迹基底，构建的仿生微/纳米环境为细胞培养提供了特定拓扑结构。实验结果表明ADSCs在该基底上能模仿角质形成细胞的形态，表达角质形成细胞的特异性标记基因和蛋白，实现向角质形成细胞样细胞的分化。通过原子力显微镜（atomic force microscope， AFM）和场发射扫描电子显微镜（field emission scanning electron microscopy， FESEM）对印迹基底进行表征，证实其能精确模拟细胞膜表面形态，从纳米到宏观尺度提供细胞“指纹”并诱导细胞分化。运用分子动力学模拟后，发现细胞核几何形状的改变会影响染色质纤维排列，进而调控基因的表达，为解释细胞形状影响干细胞分化的机制提供理论依据。

### 2.4 生物传感

近年来全细胞印迹技术在生物传感领域大放异彩^［41］^。基于全细胞印迹的生物传感器通过整合物理形貌匹配与化学特异性识别双重机制，实现了对目标细胞的高效捕获与检测^［42］^。例如，在患者疾病的早期诊断和预后中，全细胞印迹生物传感器可以在低丰度生物标志物样本中实现快速而精准的诊断。与传统生物传感器相比，其具有优越的检测灵敏度和特异性，在生物医学、精准医学中展现了巨大的潜力^［43］^。

Arreguin-Campos等^［44］^开发了一种新型生物传感器，通过表面印迹技术和热传导方法实现了疟疾感染红细胞的高灵敏度检测。该传感器以聚二甲基硅氧烷-氧化石墨烯（polydimethylsiloxane-graphene oxide， PDMS-GO）复合材料作为合成受体，通过仿生印迹技术精确复刻了疟原虫感染红细胞的形貌与化学特征（图5）。制备过程中，将含有成熟裂殖体的红细胞沉积于PDMS-GO薄膜表面，经热固化（65 ℃，4 h）及细胞裂解后形成与目标细胞匹配的微米级空腔（直径~4 μm），结合氧化石墨烯的高导热性后显著提升了热信号响应效率。实验结果表明，该生物传感器对低寄生虫血症（0.5%）样本的检出限达到临床相关水平，响应时间仅需20 min。在动态微流控测试中，感染红细胞的捕获导致界面热阻发生了显著的变化，效应值与寄生虫血症浓度呈剂量依赖性，且特异性测试显示其对健康红细胞的交叉反应可忽略（*p<*0.05）。该传感器在临床测试中表现优异，成功从13例患者全血样本中检出疟原虫感染细胞，检测效率超越传统显微镜检查。其模块化设计使操作更简便，尤其适合医疗资源匮乏地区使用。此外，PDMS-GO受体的化学稳定性支持重复使用（效率衰减< 8%），大幅降低了检测成本。

**图5:**
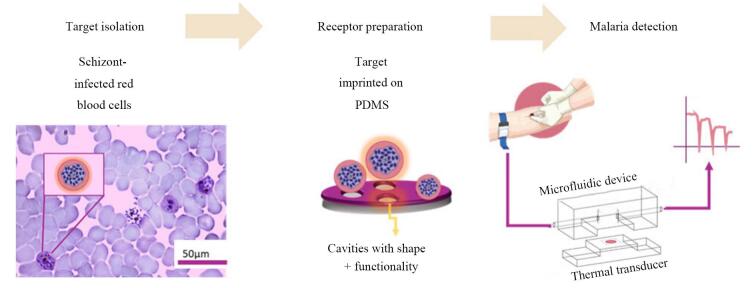
用于检测恶性疟原虫感染红细胞的全细胞热传感器示意图^［44］^

## 3 全细胞印迹技术存在的挑战

虽然目前全细胞印迹技术在国内外的应用已十分成熟，并且已经在相关研究领域取得了一定成果，但该技术仍面临着许多挑战。一方面，印迹聚合物的特异性和亲和力有待增强，其制备过程复杂、成本高昂的问题有待解决；另一方面，在实际应用于复杂生物体系时全细胞印迹技术也遭遇一系列技术难题。例如，如何确保印迹过程的高保真性、提升抗干扰能力以及增强实验结果的可重复性，已成为当前全细胞印迹技术领域的研究重点。

### 3.1 高保真性

细胞表面高度复杂并具有动态性特征，其表面分布着丰富多样的分子和微观结构^［45］^，并且这些特征会随着生理状态和所处环境的改变而发生变化^［46］^，这增加了精确复现细胞外微环境的难度。在全细胞印迹过程中，由于难以全方位模拟细胞天然环境与生理状态，致使形成的印迹位点可能与细胞实际识别位点存在偏差。此外，细胞培养和处理环节中的细微差异（温度、营养条件、培养时间）^［47-49］^，均可能对细胞表型和表面分子的表达产生影响，进而干扰印迹的准确性。当前，现有技术手段在实现全细胞印迹高保真性方面仍有很大的提升空间。后续需深入探究细胞生物学特性，持续优化印迹技术工艺，攻克上述难题，推动全细胞印迹技术在精准医疗、生物检测等领域实现可靠应用。

近期，Chen等^［50］^通过固定的细胞制备模板，将聚丙烯酰胺/壳聚糖水凝胶作为基质（60 ℃，2 h，因细胞已被固定，温度的升高对细胞生理状态无影响），借助化学交联将环状多DNA修饰到水凝胶表面，开发了一种全细胞印迹双网络水凝胶（图6）。在细胞去除后，细胞结构得以在水凝胶上保留，形成由多孔结构和纳米结节组成的层次表面。从电镜图像可以明显看出，其表面微观结构呈现出与目标细胞相匹配的特征，且随着壳聚糖/丙烯酰胺比例的调整，结节大小和多孔结构的孔径发生变化，在壳聚糖/丙烯酰胺的比例为3∶5时，水凝胶的多孔结构向不规则形态转变，这一转变对细胞黏附十分有利，提高了CTC的捕获效率。这种精确的结构复制为细胞的特异性识别和捕获提供了基础，体现了全细胞印迹技术在结构方面的高保真性。

**图6:**
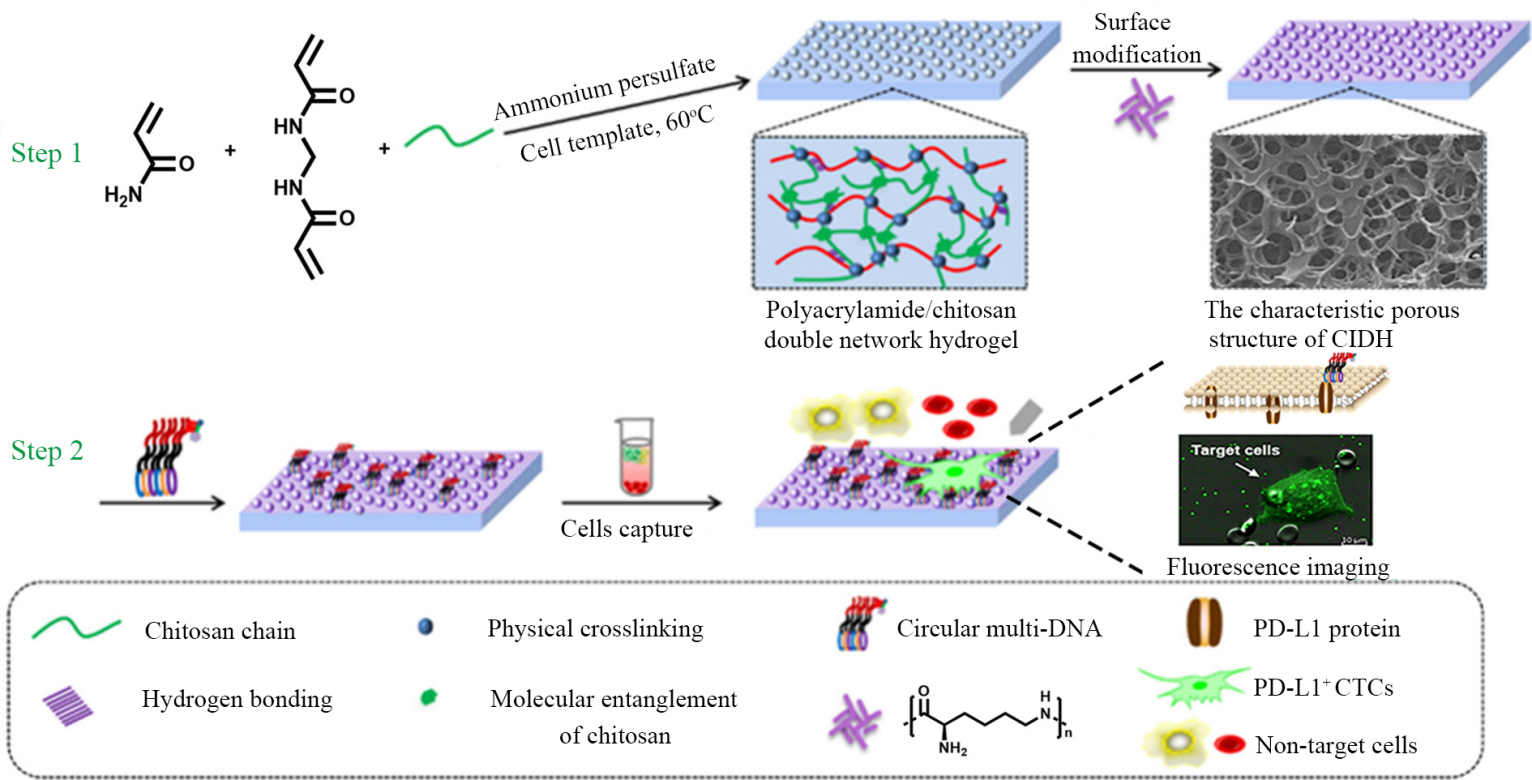
制备双网络水凝胶实现细胞的精确结构复制与捕获示意图^［50］^

Kaehr等^［51］^将化学固定后的细胞（温度变化对固定后的细胞生理状态不产生影响）浸入酸性硅酸溶液中，利用分子拥挤环境引导硅酸通过氢键和非共价作用定向沉积（图7），精准复制细胞内外纳米至微米级结构（如核孔复合体、膜褶皱）。硅网络在干燥和550 ℃煅烧中提供机械支撑，抑制毛细力与热应力导致的形变，实现超低收缩率（尺度偏差<10 nm）（图7a）。甲醇辅助渗透使硅酸均匀分布至胞内，结合缓慢缩聚机制，避免凝胶化干扰，完整保留亚细胞细节。煅烧后形成的硅骨架结构连续且致密，即便在900 ℃的热解条件下转化为导电碳材料，依然能维持原有形态，且其电导率较之前提升了20倍。相较于传统脱水法（如六甲基二硅氮烷），该策略无需复杂设备，直接以细胞固有分子排布为模板，实现从活细胞动态结构到无机材料的高保真转化（图7b）。

**图7:**
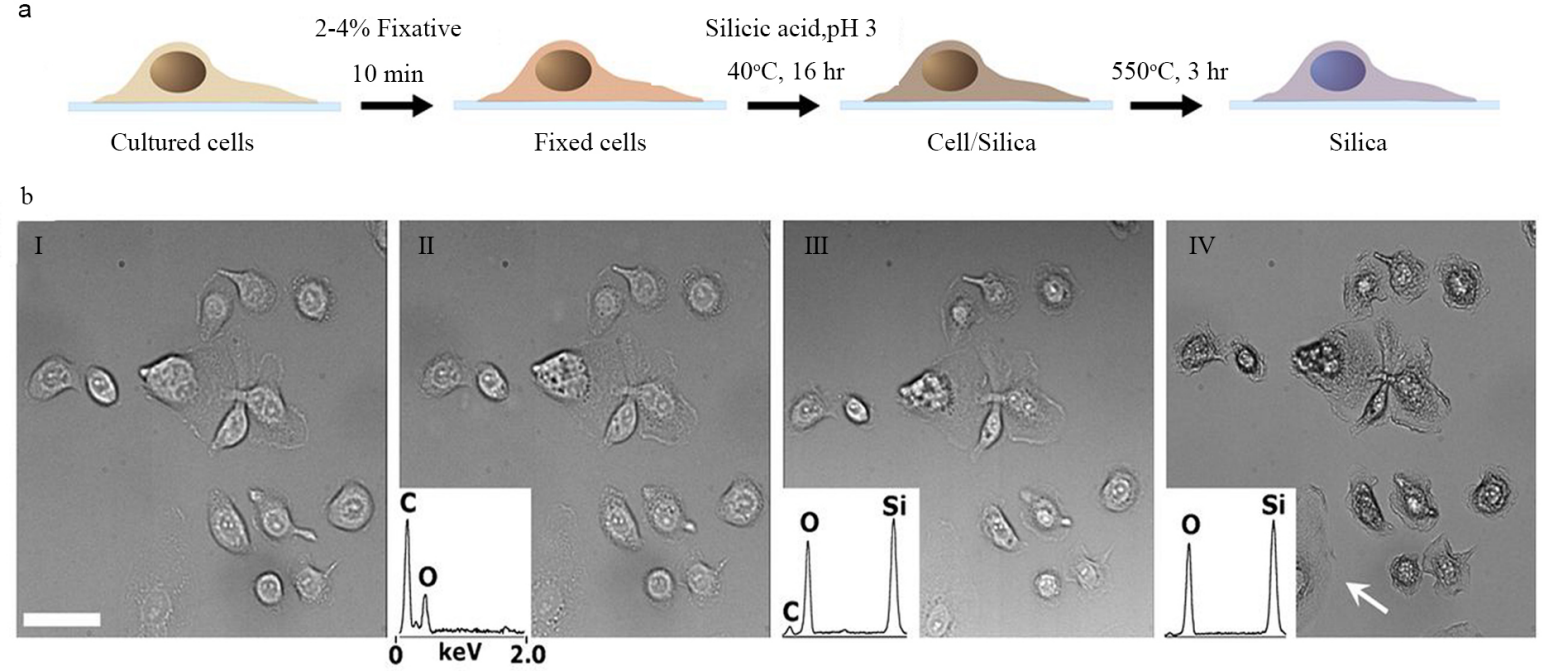
在平坦基质上培养哺乳动物细胞的硅化示意图^［51］^

### 3.2 抗干扰性

在全细胞印迹技术的发展过程中，干扰问题是限制其广泛应用的关键瓶颈。生物样本的高度复杂性是干扰产生的重要诱因，样本中大量存在的细胞或分子在形态和表面标志物等方面与目标细胞极为相似。这些“杂质”在全细胞印迹过程中，会与目标细胞竞争结合位点，严重影响目标细胞的准确识别与捕获^［2，52］^。此外，环境因素同样是不可忽视的干扰源。温度的波动、pH值的变化以及各类杂质的存在都会对全细胞印迹材料的物理化学性质造成负面影响。这些不仅改变了细胞的活性状态与印迹材料的性能，还严重降低了检测结果的准确性和可靠性。

Sun等^［53］^在CTC检测领域取得了重要进展（图8）。他们制备的聚乙二醇化硼酸酯亲和全细胞印迹的聚二甲基硅氧烷（PEGylated boronate affinity cell imprinted polydimethylsiloxane， PBACIP）为解决干扰问题提供了新的思路。在复杂的血液样本环境中，红细胞和白细胞数量庞大，严重干扰了CTC的检测。该团队通过巧妙地引入硼酸功能化氧化硅（SiO_2_）纳米颗粒来特异性结合细胞表面糖蛋白（80 ℃，2 h），根据细胞印迹技术的软光刻法制备微米和纳米级细胞印迹形貌，并对非印迹区域进行聚乙二醇化修饰，极大地降低了非特异性吸附。由于预先进行了细胞固定，所以此时温度的变化对细胞的生理状态无影响。在实际应用中，当处理乳腺癌患者血液样本（37 ℃，5% CO_2_）时，PBACIP展现出卓越的性能。其对CTC的捕获效率可达86.7%±11.5%至96.2%±2.3%，成功地减少了其他细胞的干扰，为癌症早期诊断带来了新的希望。

**图8:**
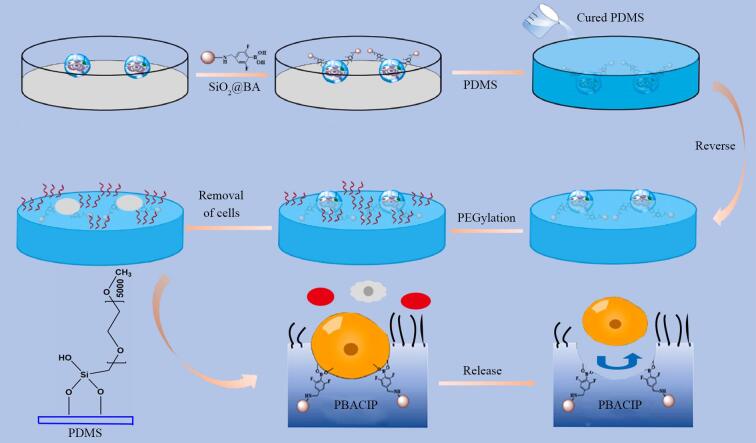
PBACIP的制造路线及选择性捕获癌症患者的CTC示意图^［53］^

此外，Gao等^［54］^研发了一种全细胞印迹海藻酸盐水凝胶（cell-imprinted alginate hydrogel， CIAH），通过软光刻技术复制了MCF-7细胞（37 ℃，5% CO_2_）的形态，全细胞印迹产生的形状互补拓扑实现了MCF-7细胞的特异性捕获（图9），并与其他细胞系（如HeLa细胞、成纤维细胞）进行对比。结果显示，CIAH界面更倾向于捕获MCF-7细胞，对HeLa细胞和成纤维细胞的捕获效率较低。经过抗体修饰后，传感器的选择性大幅提升，这归功于天然免疫识别、人工受体结合以及精确的空间匹配三者的协同增强作用，表明全细胞印迹技术在面对不同类型细胞时，能有效抵抗非目标细胞的干扰，实现对目标细胞的特异性捕获。

**图9:**
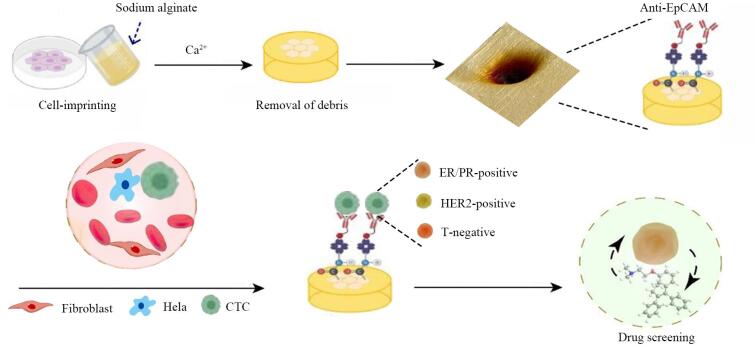
全细胞印迹海藻酸盐水凝胶用于异质性CTC的一体化诊断示意图^［54］^

### 3.3 可重现性

全细胞印迹技术的可重现性受多种因素制约。从细胞层面来看，细胞的状态差异是关键因素。不同批次细胞的生长阶段、活性以及表面分子表达情况难以完全一致，如在培养过程中，即使是相同类型的细胞，其代谢速率的细微差别也可能影响印迹效果^［2］^。在印迹材料方面，聚合物的合成过程复杂且易受多种因素干扰^［55］^。

Shahriyari等^［56］^研究制备了基于PDMS和甲基丙烯酰化明胶（gelatin methacryloyl， GelMA）的CIS（图10）。在实验过程中，对于同一类型细胞（如MCF-7细胞）的印迹操作，能够在多次重复实验中较为稳定地获得相似的全细胞印迹图案和效果，与普通基底相比，提高了细胞的代谢活性和存活率，体现出细胞在印迹底物上的生长、增殖等行为具有一定的一致性，这表明该方法在一定程度上有效克服了全细胞印迹技术在可重现性方面所面临的部分挑战，为全细胞印迹技术的进一步发展和应用提供了有价值的参考和实践基础。

**图10:**
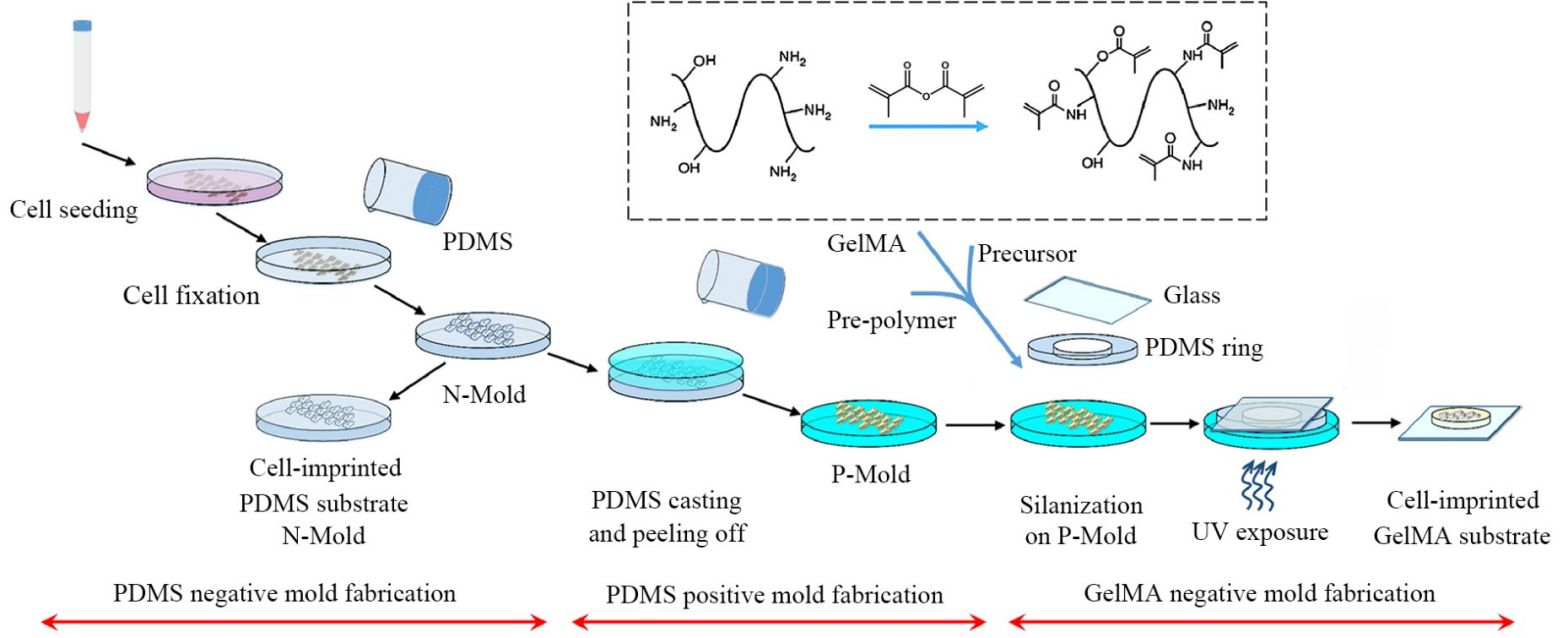
PDMS和GelMA水凝胶的全细胞印迹技术示意图^［56］^

除上述使用合适底物提高可重复性的方法外，使用人工模板也是提高可重现性的有效策略之一。Jenik等^［57］^在红细胞检测研究中，将天然红细胞经0.9% NaCl溶液洗涤离心后，用1%戊二醛溶液交联固定3 min，使其保持稳定的双凹圆盘结构；随后将固定化的红细胞单层地铺展在玻璃基板上作为初始模板，浇注PDMS预聚物并真空脱泡，室温固化48 h后超声剥离，获得具有红细胞负形结构的PDMS模具；接着在该PDMS模具表面涂覆含0.5 mol/L双酚A的环氧树脂（SU8-2025），经65 ℃预烘、紫外光固化（UV， 366 nm，4 min）和95 ℃后烘处理，最终制得具有与天然红细胞完全一致几何形貌的人工模板。整个制备过程通过化学交联固定、软刻蚀复制和热固化成型3个关键步骤，确保了模板在形貌尺寸和表面化学特性上的高度一致性。实验采用AFM对人工模板进行表征，确认其成功复制了天然红细胞的表面特征。这种标准化制备方法有效解决了直接使用天然红细胞时存在的个体差异和形变问题，高度确保了其可重现性，为后续测试奠定基础。

全细胞印迹技术尽管面临着提升高保真性、抗干扰性和结果可重复性等挑战，但通过优化材料、改进方法及规范流程，研究人员已取得显著的突破。如今，基于全细胞印迹技术已发展出众多应用成果（表1），展现出了巨大的发展潜力，不断推动着生物医学领域的进步。

**表1:** 全细胞印迹技术在生物医学领域的应用

Application	Method	Samples	Advantages	Ref.
Rare cell capture	artificial antibody replica imprinting	SMMC-7721	high efficiency， low cost	［^17^］
	stamp imprinting	MCF-7	fixed-point release， high efficiency	［^33^］
	artificial antibody replica imprinting	MCF-7	real-time fluorescence detection	［^50^］
	artificial antibody replica imprinting	SKBR-3	high specificity， efficiency	［^53^］
	artificial antibody replica imprinting	MCF-7	capture， analyze， respond to drugs all in one	［^54^］
	artificial antibody replica imprinting	SMMC-7721	high efficiency， selectivity	［^30^］
	artificial antibody replica imprinting	SMMC-7721	high efficiency， selectivity	［^31^］
	artificial antibody replica imprinting	SMMC-7721	antibody-free hydrogel	［^32^］
Single-cell sorting	artificial template imprinting	HL60	high selectivity	［^19^］
	sacrificial layer imprint method	AsPC-1， 4T1， RBL-2H3， erythrocyte	stable function， low cost， simple operation	［^51^］
	artificial antibody replica imprinting	HLE	high specificity， low cost， easy to operate	［^38^］
Cell culture	artificial template imprinting	rat cardiomyocytes H9C2	precise， rapid.	［^18^］
	stamp imprinting	rabbit ADSCs， chondrocytes	highly operable， high efficiency	［^39^］
	sacrificial layer imprint method	human chondrocytes	high efficiency	［^40^］
Biosensing	stamp imprinting	macrophages， MCF-7	low cost， fast， reusable	［^42^］
	film imprinting	erythrocytes	real-time monitoring， high precision	［^44^］
SKBR-3： human breast adenocarcinoma cell line； HL60： human promyelocytic leukemia cell line； AsPC-1： human pancreatic adenocarcinoma cell line； 4T1： murine mammary carcinoma cell line； RBL-2H3： rat basophilic leukemia cell line； HLE： human hepatocellular carcinoma cells； H9C2： rat cardiomyoblast cell line.				

## 4 总结与展望

全细胞印迹技术在生物医学领域具有重要的应用价值。在稀有细胞捕获中，可精准分离血液中的CTC；在单细胞分选中，能获取高纯度目标细胞；在细胞培养时，通过模拟微环境提升培养效率；在生物传感时，实现对细胞信号分子的灵敏检测。然而，该技术也面临着如高保真性差、抗干扰性差、可重现性差等多重挑战。

此外，全细胞印迹技术在临床转化中也面临瓶颈，其一在于技术标准化不足，其制备流程、成像和分析方法缺乏统一的规范，导致不同实验室或研究间的结果难以复现和比较，严重限制了技术的推广；其二，临床样本（如肿瘤组织）的高度异质性进一步增加了技术应用的难度，全细胞印迹可能因为无法全面捕获样本的复杂性，导致信号解读偏差，使假阳性或假阴性风险上升。

鉴于当前全细胞印迹技术存在的挑战和面临的瓶颈，其自身发展方向主要涵盖：（1）利用聚合技术改善印迹材料与模板细胞的界面相容性，有效提升二者的贴合性能；（2）通过全细胞印迹技术与其他细胞识别技术的协同，提高全细胞印迹材料的识别特异性；（3）深入研究材料与细胞的相互作用机制，最大程度减少对细胞生理功能的影响，确保检测对象及干细胞分化的活性与完整性；（4）将3D打印技术与全细胞印迹技术融合，进一步推动全细胞印迹技术在生物医学领域的广泛应用和创新发展。通过不断推进这些方向的研究与创新，有望实现全细胞印迹技术对细胞层面的高效精准分析与调控，在疾病早期筛查、个性化医疗等方面发挥关键作用，为生物医学领域带来新的突破与发展契机。
